# Policy Trap and Optimal Subsidization Policy under Limited Supply of Vaccines

**DOI:** 10.1371/journal.pone.0067249

**Published:** 2013-07-01

**Authors:** Ming Yi, Achla Marathe

**Affiliations:** 1 Network Dynamics and Simulation Science Laboratory, VBI, Virginia Tech, Blacksburg, Virginia, United States of America; 2 Department of Economics, Virginia Tech, Blacksburg, Virginia, United States of America; 3 Department of Agricultural and Applied Economics, Virginia Tech, Blacksburg, Virginia, United States of America; Northwestern University Feinberg School of Medicine, United States of America

## Abstract

We adopt a susceptible-infected-susceptible (SIS) model on a Barabási and Albert (BA) network to investigate the effects of different vaccine subsidization policies. The goal is to control the prevalence of the disease given a limited supply and voluntary uptake of vaccines. The results show a uniform subsidization policy is always harmful and increases the prevalence of the disease, because the lower degree individuals’ demand for vaccine crowds out the higher degree individuals’ demand. In the absence of an effective uniform policy, we explore a targeted subsidization policy which relies on a proxy variable instead of individuals’ connectivity. Findings show a poor proxy-based targeted program can still increase the disease prevalence and become a policy trap. The results are robust to general scale-free networks.

## Introduction

Much work has been devoted to the observation that voluntary vaccination is inefficient because of the free-riding problem and perception of risk associated with the vaccines [1]–[3]. Voluntary vaccination is also ineffective when there is a limited supply of vaccines available and there is no priority given to sub-populations such as hospital-related employees, students, grocery workers and other socially active individuals, who are more likely to contract and transmit the disease because of their positions in the social network. These results have been often mentioned in studies that assume homogeneous mixing of population and thus call for the necessity of government interventions. The problem becomes even more acute [4], [5] when a social network with heterogeneous connectivity is considered [6]–[11]. For a sufficiently heterogeneous social contact network, any non-degree-oriented policy becomes inefficient and the government should intervene by vaccinating individuals with the highest degrees first [12], [13]. However, although proving efficient theoretically, the optimal policy mentioned above is generally unlikely to be implemented in reality because (i) the government cannot discover the network degree of each individual and (ii) it cannot provide preferential treatment to individuals based on their levels of connectivity. Hence the government has to use alternative interventions such as subsidy policies.

The goal of the present work is to investigate whether a uniform or targeted subsidy policy will work under realistic circumstances. We adopt a susceptible-infected-susceptible (SIS) model on a Barabási and Albert (BA) network and add into the model a decision rule of voluntary vaccination for individuals. Our context differs from most others in the following respect. First, the government does not have the authority to decide which individuals get vaccinated, but can rather choose a subsidization policy which could incentivize (some) individuals to do so. Second, the vaccine is available in limited supply. Third, individuals are aware of their own connectivity degrees and make decisions (partly) based on this information, i.e. individuals who have a high number of social contacts are more willing to get vaccinated, this could either come from the fact that these individuals realize their risks of getting infected are relatively high or result from communications on the disease prevalence with others through their social connections [5], [14].

## Analysis

A susceptible-infected-susceptible (SIS) model [15] is used in this research. In this model, the nodes represent individuals and links stand for the social contacts through which a disease can propagate. Each individual chooses whether or not she would like to take the vaccine. Once an individual has successfully taken the vaccine, she becomes immune to the disease forever. In other words, we assume perfect efficacy of the vaccine and lifelong immunity.

We assume a closed model with no births or deaths. For the non-vaccinated nodes, we assume there is no acquired immunity after the recovery from an infection and hence their health status continuously goes through the cycle of susceptible-infected-susceptible. Each non-vaccinated and susceptible node is infected with rate 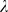 if she is connected to one or more infected nodes on the graph. Each infected node becomes cured and susceptible again with rate 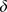. Without loss of generality we take the unit recovery rate, 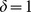, throughout this paper.

The graph is assumed to take the form of Barabási and Albert (BA) model [6], which is a classic scale-free network. The BA network is constructed through the following steps. We start with a few disconnected nodes; then in each step a new node is added to the existing graph, with 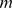 new links connecting her and the old ones. Moreover, the probability an old node 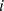 would be connected to the new node is given by 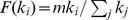, where 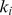 denotes 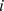
’s connectivity (degree). This implies higher the degree, the easier it is for an old node to attract connections from new individuals; this is also known as the “preferential attachment model”. We assume large network size N and take 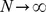 throughout this paper. After normalization and continuous 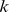 approximation, we have the degree distribution 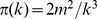 and 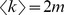, where 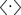 denotes the expectation. Let 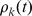 be the fraction of infected individuals (disease prevalence) within connectivity-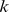 group at time 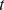.

For an individual with degree 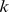, her probability of getting the vaccine is assumed to be a function of 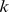 and vaccine price 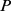, 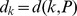. More specifically, we take 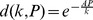, where 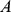 is a constant parameter. We take this form of (probability) demand function for tractability of analysis as well as the following merits:

• 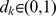 always holds, so 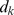 is properly defined as a probability.

• 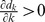, the higher the connectivity degree, the higher the willingness of a node to get vaccinated. When 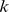 approaches 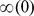, 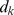 approaches 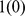.

• The price elasticity of demand for an individual with connectivity 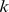 is 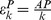, which is decreasing in 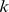: When other things are fixed, individuals with a higher degree exhibit lower price elasticity of demand i.e., the higher the degree of an individual, the less sensitive the individual is to changes in price. This property conveys the ideas and observations we make about the higher-degree individuals who are likely aware of their risk of exposure.

Each individual decides whether or not to take the vaccine at 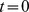, vaccines are no longer available after the disease has started propagating. However, not all demand can be satisfied given the limited supply of vaccines. The supply level is fixed at 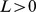, and 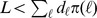 holds for all 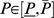, where 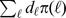 stands for the total demand of the population and 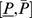 is the proper domain in which an authority can set the price of vaccine through subsidies. The limited stockpile of vaccines is distributed to individuals on a first-come first-serve basis. We thus define the fraction of immunized individuals within connectivity-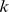 group as
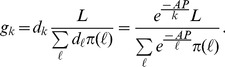
(1)


Within connectivity-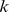 group, all individuals are treated identically since they share the same price, the same degree 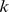, and the same probability demand function 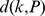. So with 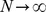, the law of large numbers tells us 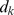 is also the fraction of individuals who are willing to take vaccine *within* connectivity-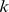 group. We also recall some of these individuals who wanted to purchase the vaccine could not do so due to the insufficient supply; multiplying this fraction by the probability of getting vaccinated thus leads to the immunized fraction within the group.

Now we can track the evolution of disease prevalence 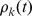 for any given 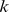 through mean-field equation [13], [16], [17]

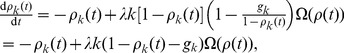
(2)where the first term on the right-hand side reflects the unit recovery rate assumption made above. The second term measures the probability a healthy node in this group becomes infected through contacts with others. Given the vaccine works perfectly, we need to remove the subgroup of immunized individuals from consideration and product 
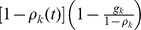
 gives us the fraction of susceptible nodes within the group. For each susceptible node, her probability of getting infected is proportional to the spreading rate 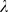 and the number of links connecting her to an infected node 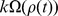, where 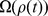 is the probability any given link connects to an infected node. More specifically, 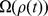 is calculated as
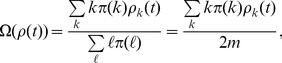
(3)where the second equality is because 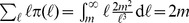 for 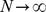. We focus on the stationary state of the system; imposing condition 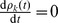 on equation (2) gives us expressions for stationary within-group fractions of infected nodes for all groups i.e
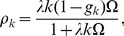
(4)where
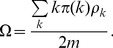
(5)


## Results

In this section, we first show why a uniform subsidy policy could make things even worse, then investigate the effects of a targeted policy, and check the robustness of our results on general scale-free networks.

### Uniform Subsidization

#### Persistence of the disease

Substituting equation (4) into (5) and imposing continuous 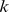 approximation give us.
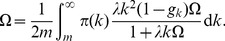
(6)


Obviously equation (6) admits a trivial solution 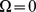. To check the existence of a non-trivial solution, denote by 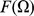 the right-hand side of (6), we have 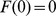, 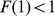, and
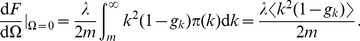
(7)


Define 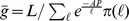, then from equation (1) we have 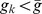 always holds, this in turn gives us
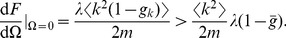
(8)


Since 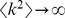 in the BA network, we have 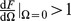, this suggests equation (6) admits a non-trivial solution 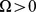. To see this non-trivial solution is unique, just check that 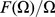 is strictly decreasing in 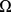.

With 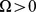, we have 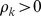 for each 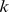, and the disease always persists. The persistence of disease stems from three main aspects of our model: *a scale-free network*, *voluntary vaccination*, and *limited supply*. With a homogeneous population, it suffices to impose a uniform vaccination policy to eliminate the disease as long as the limited supply is above the herd immunity threshold [3], [13], [15]. Even for a general network that is not as heterogeneous as a scale-free network, an infinite-size population consisting of fraction 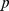 people with degree 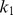 and fraction 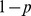 people with degree 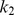, the disease could also be eliminated through herd immunity under a limited vaccine supply and voluntary vaccination. In the case of involuntary vaccination, the disease could be controlled in the stationary state in a scale-free network given a limited supply of vaccines, if the government was able to identify all the highest degrees individuals and had the power to vaccinate them [12], [13]. With an unlimited supply, 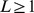, the government can simply set a price low enough so that 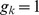 for all groups and the disease gets eliminated. Our results suggest as long as the vaccine is insufficiently supplied, individuals take the vaccine voluntarily, and the social network is scale-free, given any spreading rate of the disease, and any price level of the vaccine, the disease always persists.

#### Crowding out effect for uniform subsidy policies

Given that the disease cannot be eliminated, we examine if the authority can help decrease the prevalence of the disease through subsidy programs. Subsidization is believed to be helpful in scenarios where the supply is not a problem. But if supply is insufficient, subsidization may be considered to make the vaccine available to a broader class of people. This research shows a uniform subsidy policy may be a bad idea since it could have the unintended effect of increasing the prevalence of the disease.

Under the uniform subsidy policy, each dose of vaccine is subsidized by an equal amount, so we can just treat this as a price drop for every individual. After continuous 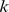 approximation we have
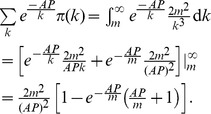
(9)


Now define 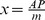, inserting (9) into (1) yields
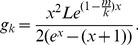
(10)


Define 
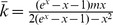
, it is then straightforward to check from equation (10) that 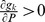 for all 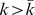 and 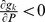 for all 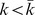: Because of the price decline, fewer high degree individuals successfully get vaccinated and more low degree individuals manage to do so, where 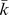 is the threshold for the high degree-low degree dichotomy. To understand this, recall the price elasticity of demand is decreasing in degree 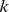, which implies for the same amount of price drop, the magnitude of response of the low-degree nodes is greater than that of the high-degree ones. As a result, the proportional increase in demand for vaccines by the low-degree individuals is greater than that of high-degree individuals. Combining this result and the condition that the stockpile of vaccines is limited and is distributed on a first-come, first-serve basis, a part of high-degree individuals’ demand thus gets *crowded out* by the low-degree individuals.

#### Policy trap

It has been proven optimal to vaccinate all high degree individuals under limited supply [12], [13]. However the uniform subsidization policy seems to push the society even farther away from the optima by resulting in more low degree individuals getting vaccinated than high degree ones. To further examine this argument, we define the prevalence rate of the disease in the stationary state as
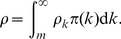
(11)


After inserting equations (6) and (10) into it, equation (11) gives us 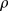 as a function of price 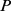. Because 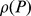 does not admit a close form, we use numerical examples to show their relationship.

**Figure 1:**
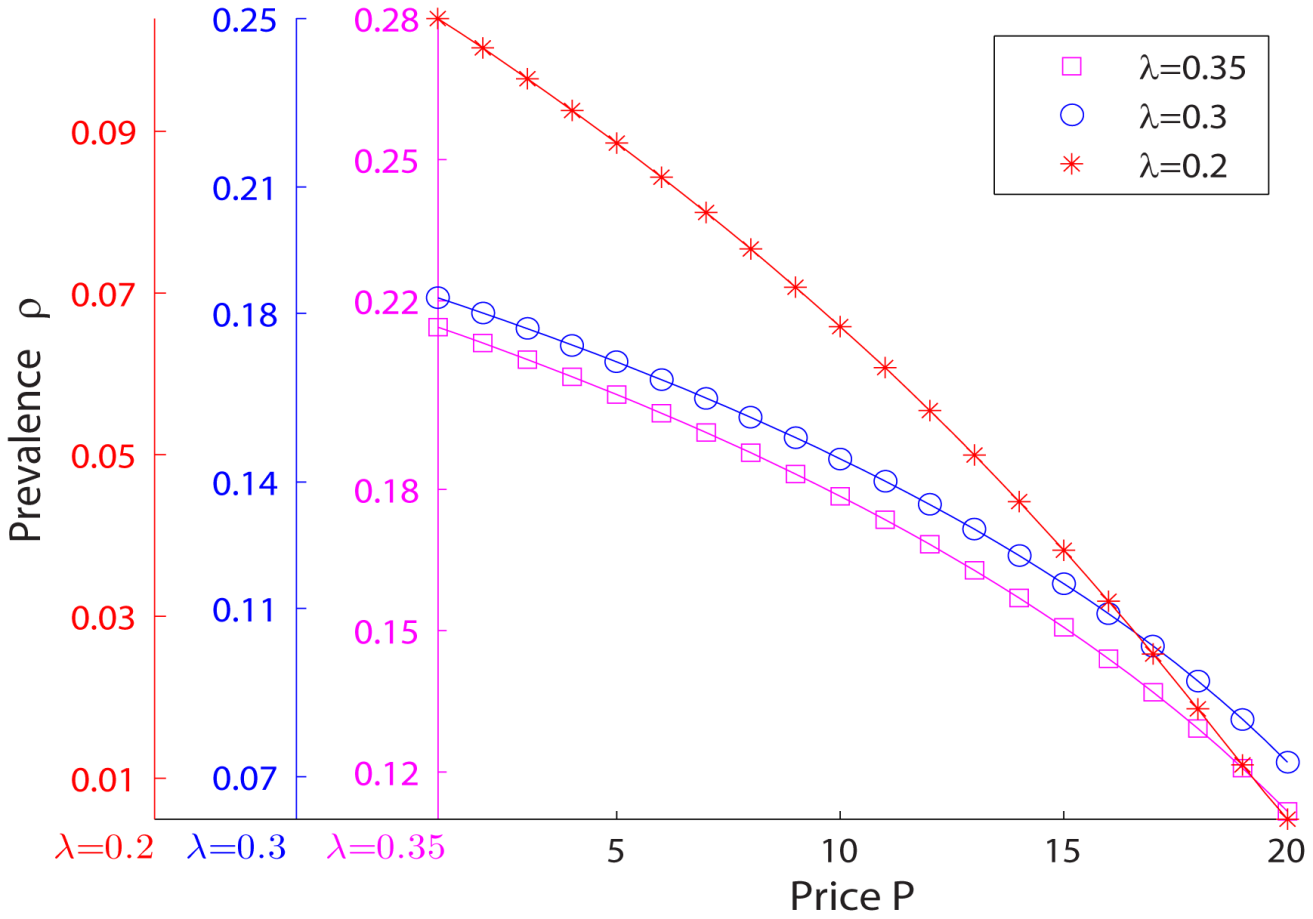
Policy trap for uniform subsidization policies. With 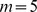, 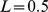, 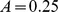, 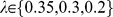, and 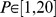.

As shown in Figure 1, in all example cases, we have 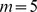, 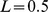, 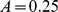, 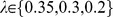, and 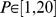. We calculate the prevalence rate 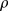 for each 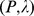 pair. For all cases, the disease always persists in the stationary state, and given other conditions fixed, the lower the spreading rate 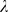, the lower the stationary state prevalence rate 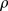. Furthermore, given any spreading rate 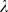, the stationary prevalence rate is decreasing in 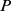. The uniform subsidization policy now becomes a *trap* because a subsidy in price meant to stimulate the usage of vaccines and drop the prevalence of the disease, actually results in a higher prevalence of the disease in society.

Under insufficient supply, vaccine is indeed a scarce good for the society. In the absence of any government intervention its distribution is regulated by price. In our scenario, individuals with a higher willingness to get the vaccine happen to be the ones who have higher connectivities (degrees), and the latter should be given priority when considering an optimal policy. In other words, the individual interests coincides with the public interest. For this reason, a higher vaccine price determined by the free market could be more efficient since the scarce resource is more likely to be (automatically) distributed to individuals from whose vaccination the society benefits most.

**Figure 2:**
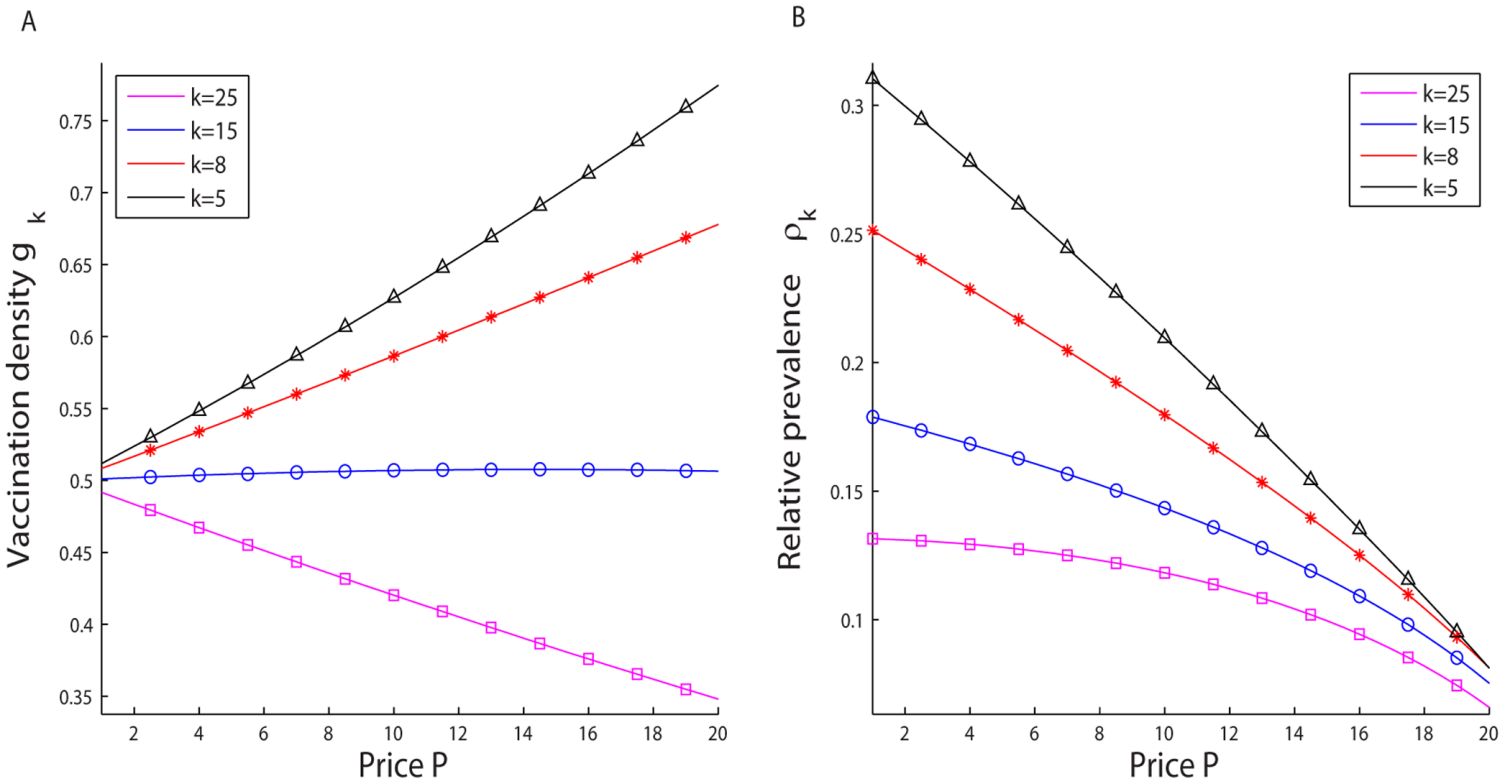
Crowding out effect and its effect on prevalences within different groups. **A.** Within-group fraction of vaccinated individuals, for different degree groups, as a function of price. **B.** Relationship between within-group prevalence rate and price, for different degree groups. In all examples, 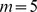, 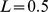, 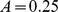, 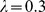, and 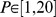.

On the other hand, under a uniform subsidization policy, as discussed above and shown in part A of Figure 2, the price drop causes an increase in vaccination rate among low-degree groups and leads to a lower vaccination rate among high-degree groups–fewer high-degree individuals get vaccinated. And since each susceptible individual is more likely to be linked to the high-degree ones, drops of vaccination rate in high-degree groups in turn lead to higher prevalence rates in all groups. As shown in part B of Figure 2, with a price drop, 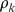 increases for each group. A uniform subsidization policy weakens the coincidence between private interests and public benefit, distorts the market, and leads to a higher disease prevalence.

### Targeted Subsidization and Proxy Variables

Knowing a uniform subsidization policy results in a worse outcome, the government may consider non-uniform subsidies, e.g. subsidize a targeted subgroup of the population. Theoretically, it is optimal to subsidize individuals with the highest degrees–set a degree threshold 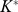, and then subsidize individuals 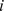 with 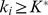.

However, while considering a proper subsidization policy, the government needs to follow some social protocols that guide the spending of tax payers’ money. These protocols are often based on some notion of equality, fairness, health status and demographics of the population. For example, the federal government frequently prioritizes the vaccination of pregnant women, immune-compromised individuals, infants and elderly because they are either at a higher risk on contracting the disease or suffer more from being ill. The optimal policy based on the connectivity of the individual does not accord with any typical social norm. The government cannot subsidize high degree individuals just because they have a relatively high degree. Moreover, it is not easy to determine a person’s degree. It is possible to identify some *proxy variables*, e.g. demographics such as age, income, etc. that correlate well with degree. The government can subsidize targeted individuals based on characteristics in the *proxy variables*, in order to control the disease without appearing biased towards a group of individuals. We argue in this section that it is possible for the government to achieve its goal if it chooses an appropriate proxy variable. For it to be the right proxy variable, it should be well rank correlated with connectivity.

Without the loss of generality, we use 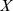 to represent the proxy variable used by the authority for setting the subsidization policy. 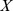 could either be a demographic variable or a function of demographic (multiple) variables. For example, if the authority wants to subsidize based on income level, we can set 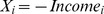 for each individual 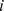; if the authority wants to subsidize based on the degree of illness, we have 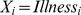. If the subsidy is based on age so children and elderly can be protected, we can set 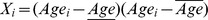, where 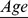 and 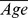 are the thresholds of the youth and senior subgroup, respectively. In all scenarios, there exists a threshold 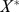 such that an individual gets subsidized if and only if 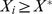.

To check the “closeness” between proxy 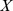 and the connectivity degree 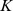, we adopt Spearman’s rank correlation coefficient [18]. The rank correlation, 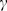, uses rankings to calculate the correlation and measures the strength of monotone association between 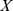 and 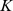. In other words, if we rearrange both 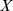 and 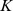 into ascending permutations, 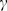 represents how well the two resulting rankings are matched. 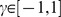 always holds. If 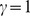, the two rankings are perfectly matched: As long as we have 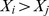 for some individuals 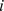 and 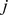, we must also have 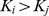. For 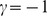, 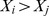 implies 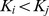.

The initial vaccine price is 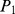, and the authority sets a threshold 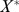 such that any individual 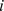 with 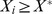 is subsidized to buy vaccines at price 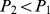. Denote by 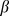 the ratio of subsidized individuals among the whole population, i.e., 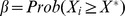. Next step is to find the fraction of subsidized individuals within each connectivity degree group. For simplicity and tractability of analysis, we define the fraction of subsidized individuals in each connectivity group as a function of rank correlation 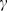 and 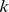, 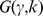. Specifically, we use the form
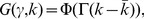
(12)where 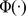 is the cumulative distribution function of a standard normal distribution,
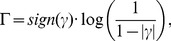
(13)and 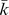 solves



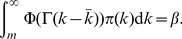
(14)We take this special form of 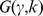 because of the following merits. Equation (12) ensures 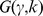 locates between 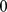 and 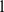. In equation (13), 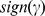 is 1 (−1) if 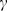 is positive (negative), and equals 1 if 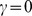. With positive (negative) 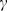, the larger the degree 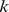, the higher the fraction of subsidized individuals within the connectivity-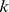 group. What is more, with 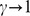 (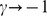), we have 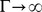 (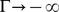), which in turn implies 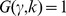 for all (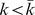) (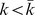). If the two rankings are perfectly (either positively or negatively) matched, the targeted subsidization policy on 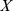 corresponds to a threshold subsidization policy on 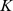. And finally, this threshold 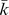 is defined in equation (14). With 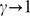, (14) can be rephrased as
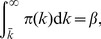
(15)so when the proxy variable 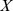 and connectivity degree 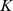 are perfectly rank correlated, a targeted subsidization policy on 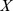 is equivalent to a subsidization policy targeted on 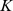. Note 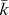 is actually an implicit function of 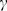.

Now for the subsidization policy targeted on 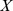, within connectivity-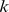 group, the fraction of individuals who are willing to buy vaccine becomes
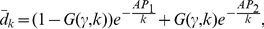
(16)where the first term on the right-hand side denotes the (probability) demand by the individuals who do not get subsidized, and the second term measures the demand of individuals who are subsidized because 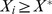 holds. Similar to what was done earlier, we insert equation (16) into (1) to get the fraction of vaccinated individuals within groups



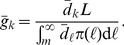
(17)Finally, replacing 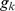 by 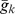 in equations (4), (6), and (11) gives us again numerical solutions of disease prevalence for difference cases.

**Figure 3:**
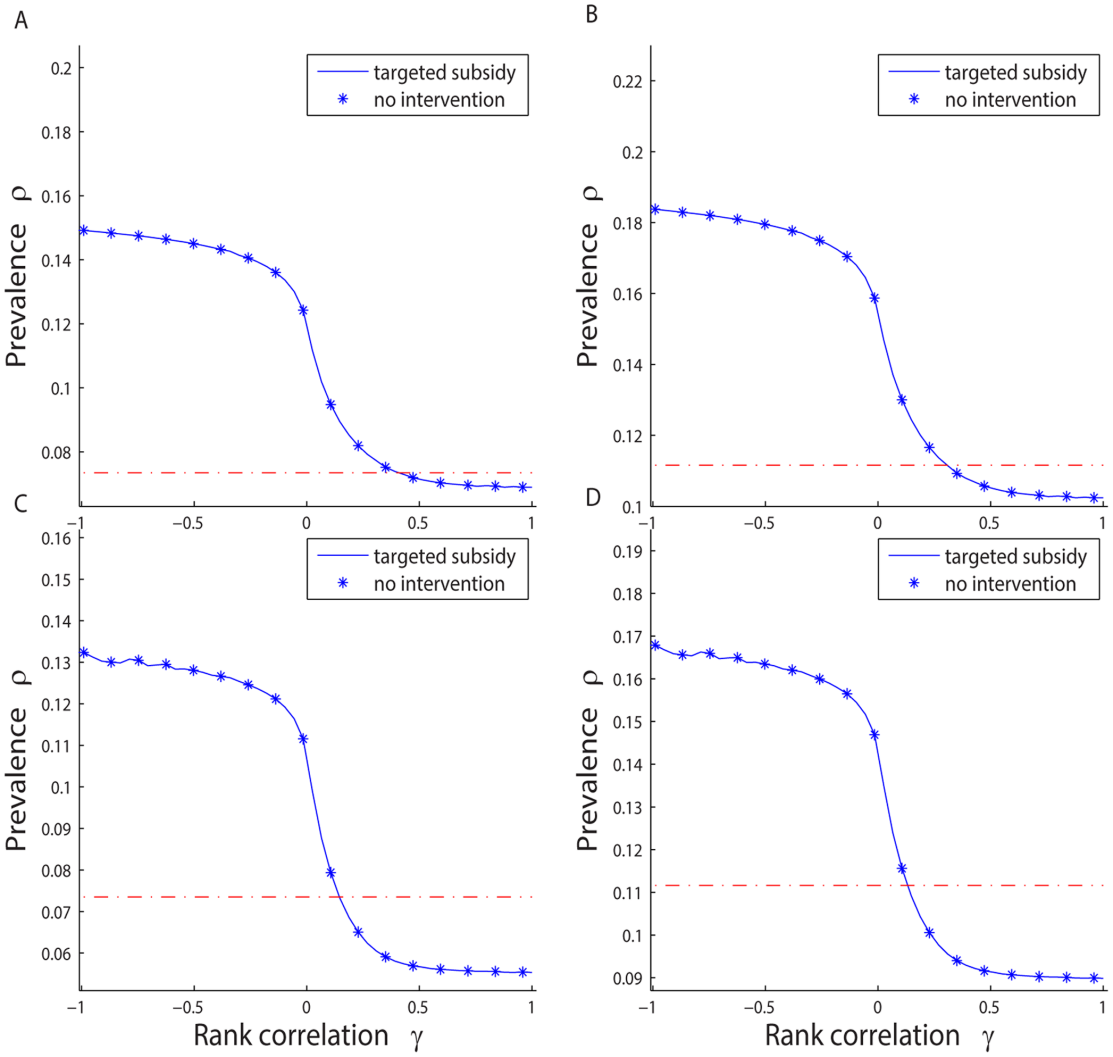
Policy trap for targeted subsidization policies, and their comparison with intervention-free cases. **A.** 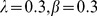. **B.** 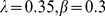. **C.** 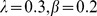. **D.** 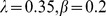. In all cases we have 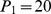, 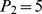, 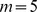, 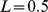, 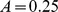, and 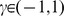.

We construct example scenarios to analyze the effect of a targeted subsidization policy which uses a proxy variable and compare it with the base case i.e., the case where no interventions are imposed by the government. We set 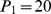, 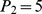, 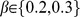, 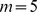, 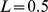, 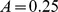, 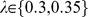, and 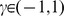. Figure 3 shows for all examples under targeted subsidization, generally speaking, the higher the rank correlation coefficient 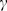, the lower the prevalence 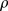 in the stationary state. This intuitively makes sense since the higher the rank correlation, the better matched is the proxy variable with the connectivity, and the more stimulation is given to the high-degree individuals. Finally, the prevalence rate is lower because vaccines tend to be distributed to the more important nodes.

Figure 3 also shows the prevalence levels for the base cases of no intervention, where all individuals face the same price 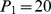. In all cases, a targeted subsidization outperforms the base line only for large enough 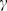 values. For instance, in case 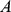, the prevalence under subsidization is lower than in base case if and only if 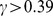.

Although a uniform subsidization policy proves counter productive, it may still be beneficial to uniformly subsidize a subgroup of individuals based on a proxy variable. However, as shown above, it is crucial to have the proxy variable chosen in such a way where it truly represents the degree. If a proxy variable is not or could not be properly chosen due to the fairness concerns or other constraints, the targeted subsidization policy could also become a policy trap, just like the uniform subsidization policies.

### General Scale-Free Networks

So far we have illustrated our results based on the BA network with an infinitely large size of the population. Recall the BA networks is a special case of a wider class of scale-free networks that are widely observed in the real world, e.g., social contact networks through which epidemics propagate. For this reason, we would like to check the robustness of our results with general scale-free networks.

For a general scale-free network in which an individual possesses at least 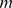 links, we have the density for individuals with 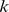 links as 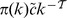, where 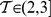 and 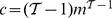 is a normalizing constant that makes the distribution well defined. With similar treatments and logic used above, we still get a unique non-trivial solution for the stationary state. Furthermore, we find the policy trap could still arise if the authority is imposing an inappropriate policy or utilizing a bad proxy variable for targeted subsidization, as shown in the following two figures.

**Figure 4:**
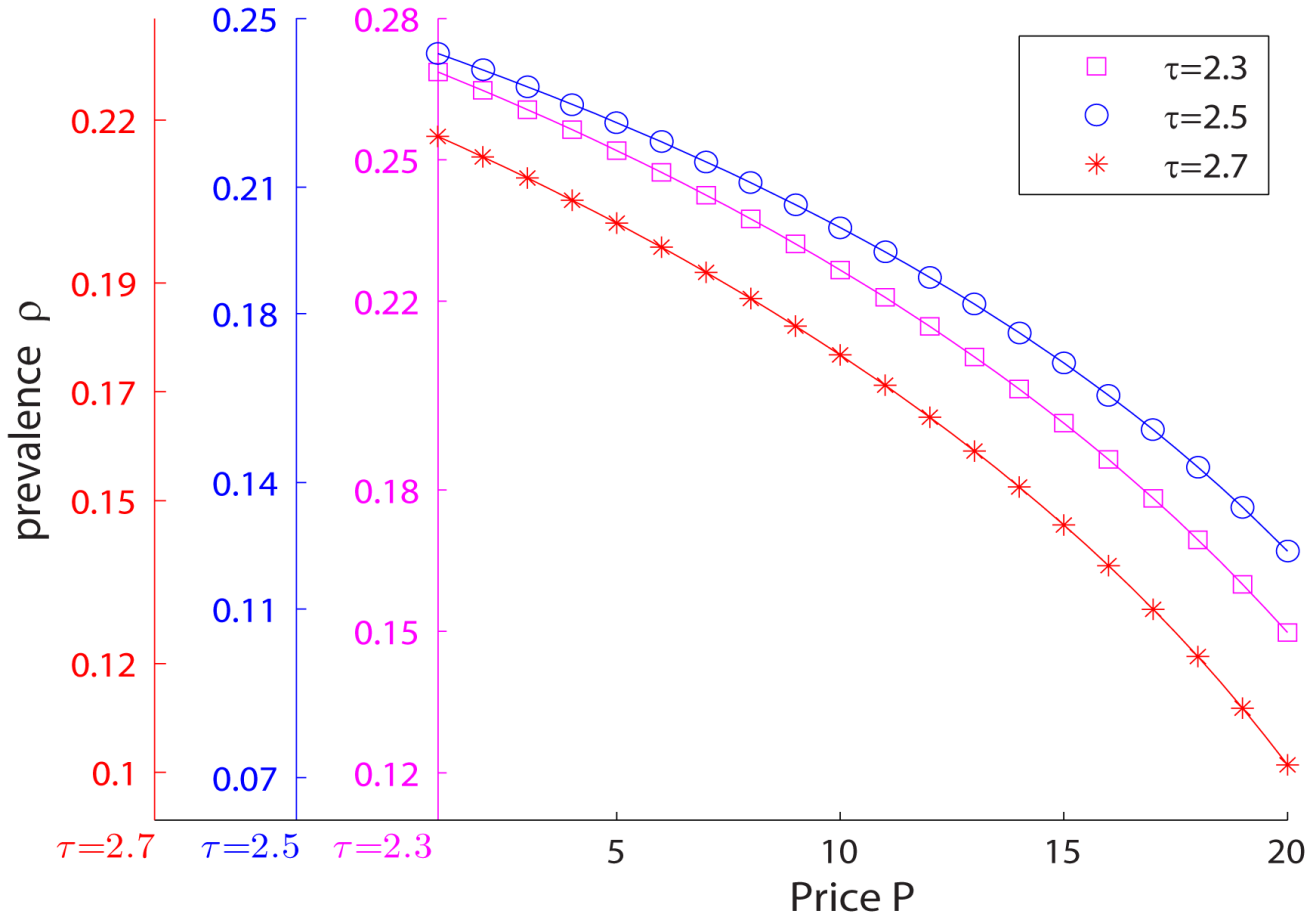
Policy trap for uniform subsidization policies and general scale-free networks. With 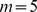, 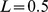, 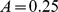, 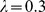, and 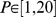.

In Figure 4, we again consider the effect of a uniform subsidization policy while keeping other factors unchanged from the examples in Figure 1. As we can see from the results, for any parameter 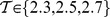, the stationary state prevalence is still decreasing in price (the prevalence of disease is much higher than the BA network case), which in turn suggests a uniform subsidization policy still does not help and the policy trap results are robust to the general scale-free network class.

**Figure 5:**
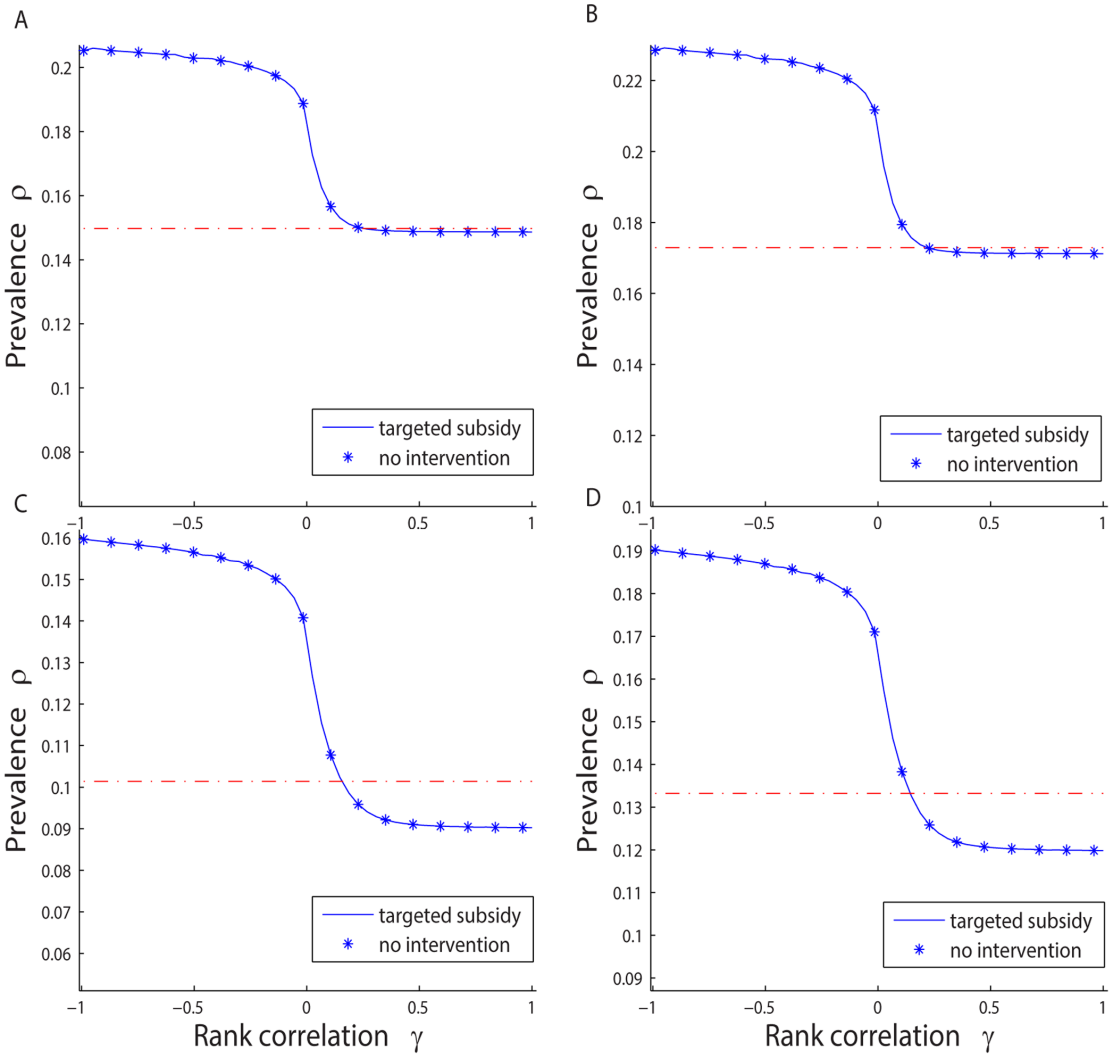
Policy trap for targeted subsidization policies, and their comparison with intervention-free cases, for general scale-free networks. A. 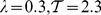. B. 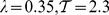. C. 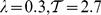. D. 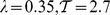. In all cases we hold 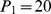, 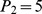, 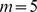, 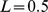, 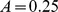, 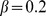, and 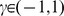.

We also check the performance of the targeted subsidization policies on general scale-free networks. As shown in Figure 5, we consider four example cases “close” to those in Figure 3. We hold the parameters unchanged from the previous examples so the differences are mainly because of the introduction of general scale-free networks. Once again, the results are similar to the BA network case. Given any 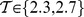, and any rank correlation 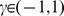, the higher the transfer rate 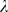, the higher the stationary state prevalence rate; and given any 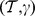 bundle, the policy trap could still occur. The targeted subsidization policy outperforms the base case only if the proxy variable is well correlated with the connectivity, otherwise intervention makes it even worse than the BA network case. The results are robust to the general scale-free networks.

Figure 5 suggests the efficacy of a targeted subsidization policy depends on parameter 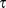 as well. As shown in example cases 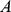 and 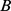, when 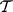 is relatively small 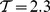, it is really difficult to outperform the non-intervention base. Even if the authority has the chance to find a perfect proxy (say 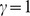 for instance), the benefit from imposing the targeted subsidization policy is almost negligible. Actually, if we consider even smaller 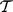, such as 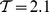, there will be no intersection between the intervention case and non-intervention base as shown in the figure, which means no matter how “good” the proxy variable is, the targeted subsidization policies always make situations worse.

The intuition behind this result is that targeted policies are most suited for networks where individuals with high connectivity constitute a relatively thin tail of the distribution, which allows these individuals to be targeted for vaccination. However, the smaller the parameter 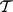, the thicker the tail of the network degree distribution. So given the same level of intervention, 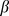, only a small fraction of the highly-connected individuals can be vaccinated, making the policy ineffective, even if the proxy is “perfect”. Eventually the potential space for improvement from the targeted subsidization policy becomes much smaller in case of scale-free networks with lower exponents, as shown in examples 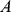 and 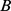. The relationship between the size of the tail of distribution and the value of parameter 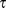 also explains why we have a lower prevalence rate in case 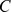 (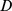) compared to that in 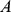 (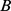), for any given rank correlation 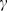 while holding all other parameters the same.

### Conclusions

This research shows that in realistic settings such as voluntary vaccination, scale-free population and limited supply of vaccines, a well intended policy of subsidization of vaccines can backfire and result in increased prevalence of the disease. A uniform subsidization meant to help the underprivileged can result in crowding out the demand of higher degree individuals by the lower degree individuals. A targeted subsidization may be implemented to reach the high degree individuals, however a justifiable demographic based proxy variable is needed to screen out the high-degree individuals.

If a poor proxy variable is selected, the targeted subsidization policy could also become a policy trap just like the uniform subsidization policy. A good proxy variable with a high Spearman’s Rank Correlation with the degree of the individuals can help the government achieve its goal of controlling the disease for a scale-free network with a relatively large exponent parameter (such as 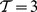 for the BA network); however, for general scale-free networks with smaller exponent parameters (such as 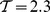), the same intervention policy may become less effective or even harmful.

This highlights the importance of understanding the structure and heterogeneity of social networks while making targeted subsidization policies. In other words, it may be inappropriate to impose the same intervention to different areas if their respective social networks are believed to differ significantly. Although the analyses here are based on a theoretical model with specific and stylized settings, future work will test the robustness of our results on realistic social networks [19], [20].
